# Teaching LLMs How ICU Physicians Approach Clinical Reasoning Through OMOP-Aligned Retrieval Improves Reasoning Across Clinical Domains

**DOI:** 10.21203/rs.3.rs-11157053/v1

**Published:** 2026-10-14

**Authors:** Miguel Contreras, Scott Siegel, Subhash Nerella, Jessica Sena, Jiaqing Zhang, Heng Sun, Hruday Tej Akkaladevi, Peiyu Lu, Jordan Rosen, Sumit Kapoor, Sasank Desaraju, Grace R. Thompson, Jacob Purcell, Michael Petrauskis, Philip KW. Hong, Meghan Brennan, Sarah Chrabaszcz, Tierra Smith, Ronnie Ren, Michel S. Kabbash, Ceyhun Haziroglu, Rushi Patel, Gabriel Gomez, Charlotte Chaiklin, Randy Leung, Kenneth N. John, Whitman Wiggins, Philip Kayser, Vincent Bird, Maria Bruzzone, Tyler J. Loftus, Azra Bihorac, Parisa Rashidi

## Abstract

Clinical decision-making relies on identifying relevant patient information to guide diagnosis and treatment, a challenge that is especially difficult in the data-dense and rapidly changing intensive care unit (ICU). Large language models (LLMs) could support this task. However, existing applications and datasets mostly emphasize surface-level retrieval or factual recall rather than the inductive and deductive reasoning clinicians practice to select and reason over decision-relevant evidence. We hypothesized that training LLMs on expert ICU reasoning could yield clinical reasoning skills that generalize beyond critical care. Here we introduce ICU-REACT, a reasoning dataset developed with 19 clinicians through a clinician-in-the-loop framework to teach LLMs to perform information retrieval and context-aware clinical reasoning in the ICU. Using ICU-REACT, we fine-tuned Clin-REACT models spanning 8B-70B parameters and three model families. Across five clinical reasoning benchmarks, Clin-REACT consistently outperformed its backbone models and open-source general-purpose and medical LLMs. Gains extended to different tasks including script concordance tests, and downstream diagnosis and treatment tasks. These findings suggest that expert reasoning supervision in critical care can improve broader clinical reasoning, although prospective evaluation is needed before real-world clinical use.

## 1 Introduction

The intensive care unit (ICU) is a fast-paced and constantly evolving environment where clinicians must make timely decisions based on large amounts of patient data. These decisions often require quickly identifying and interpreting relevant information from electronic health record (EHR) data, including vital signs, laboratory results, medications, and imaging reports [1]. Navigating these systems remains complex and time-consuming, limiting clinicians' ability to efficiently extract and synthesize decision-relevant information [2].

Large language models (LLMs) have shown potential to reduce the burden of navigating EHR data by enabling natural-language interaction with patient records [3–5]. However, existing applications remain largely focused on surface-level tasks such as retrieving information from clinical notes or extracting discrete variables. Clinical reasoning entails substantially more complex tasks. Clinicians must identify relevant data to then generate a probability-ranked set of hypotheses consistent with the available observations, perform diagnostic testing to evaluate those hypotheses, and implement patient-specific treatment plans. In this sense, identifying decision-relevant information is itself part of the reasoning process rather than a separate retrieval task. Therefore, we hypothesized that the ICU, with its dense information, clinical complexity, and uncertainty, could provide a high-yield setting for teaching inductive and deductive clinical reasoning. By learning to identify the most relevant evidence and to perform multi-layered clinical reasoning with incomplete information, models may develop clinical reasoning skills that transfer across clinical domains.

Realizing this potential requires training resources that supervise not only the final clinical answer, but also how relevant evidence is selected and used to reach it. However, existing datasets primarily target general medical question-answering [6, 7], isolated EHR retrieval tasks [8, 9], entity linking [10], consistency checking [11], or individual reasoning dimensions such as temporal reasoning in longitudinal records [12]. These resources have advanced clinical language modeling, but they offer limited supervision of how clinicians identify relevant patient information, place it in context, and use it to support a clinical decision. A similar pattern has been seen at the model level. Earlier medical LLMs were trained primarily to acquire and reproduce medical knowledge [13], with performance commonly assessed using traditional multiple-choice benchmarks [6, 7, 14, 15]. More recent medical LLMs increasingly incorporate reasoning-focused training [16–19], representing an important shift beyond factual recall. Yet their training and evaluation still rely heavily on the same conventional multiple-choice benchmarks, which provide only a limited view of whether they can identify the most relevant information and reason through realistic patient contexts.

More recent benchmarks have begun to address this gap by evaluating more complex forms of clinical reasoning. Some datasets have focused on script concordance tests that assess agreement with expert judgment under uncertainty [20], while others have evaluated broader clinical reasoning using structured cases [21, 22]. Other benchmarks have extended this paradigm to EHR-grounded reasoning in the emergency department [23], and to sequential diagnosis requiring models to iteratively gather information, update hypotheses, and select subsequent tests [24]. Importantly, a recent dataset introduced long-context ICU evaluation tasks centered on patient assessment and action recommendations [25]. Together, these efforts represent important progress toward more realistic evaluation of clinical reasoning tasks, but they are still designed primarily to assess model performance rather than teach models how to find, prioritize, and reason over clinically relevant information. This limitation highlights the need for clinician-curated datasets that can support both the training and evaluation of multi-stage clinical reasoning.

To address this gap, we introduce ICU-REACT (Intensive Care Unit Reasoning for Electronic Health Record-Anchored Decision Support Tasks), a clinician-supervised dataset designed to teach LLMs how to identify and reason over decision-relevant information in realistic ICU scenarios. ICU-REACT was developed by first creating a seed set through a clinician-in-the-loop framework involving 19 clinicians across multiple specialties. The clinician-curated seed dataset was subsequently augmented for model training using a self-instruct methodology [26, 27]. Each sample links an actionable clinical question to a patient context, a set of relevant EHR variables, and a rationale explaining why those variables are important to the decision. Clinical variables are mapped to the Observational Medical Outcomes Partnership (OMOP) Common Data Model, providing a standardized representation for information retrieval across heterogeneous EHR systems. We use ICU-REACT to develop Clin-REACT (Clinical Reasoning for Electronic Health Record-Anchored Decision Support Tasks), a family of LLMs fine-tuned to identify decision-relevant patient information and generate explicit, context-grounded reasoning connecting that evidence to clinical decisions.

We then test our broader hypothesis: whether reasoning supervision confined to the ICU can improve clinical reasoning beyond the domain in which it was learned. Clin-REACT models are evaluated on the held-out ICU-REACT test set and four independent external clinical reasoning benchmarks spanning critical care, emergency medicine, and general clinical reasoning: SCT-Bench [20], ER-Reason [23], MedR-Bench [21], and VivaBench [22]. Across this evaluation suite, Clin-REACT models consistently outperformed their backbone models and performed strongly against open-source general-purpose and medical LLMs. Notably, these gains extended beyond ICU information retrieval and reasoning to downstream diagnostic and treatment tasks in other clinical settings. These results support our hypothesis that clinician supervision in a complex, high-acuity environment can teach models skills that transfer beyond the setting in which they were trained. Together, ICU-REACT and Clin-REACT provide a framework for training and evaluating clinical LLMs not only on retrieving patient information or generating answers, but on identifying which evidence matters and reasoning over it. Prospective evaluation with practicing clinicians remains necessary to determine clinical utility, safety, and readiness for real-world use.

## 2 Results

Figure 1 summarizes the overall workflow for developing ICU-REACT, training Clin-REACT, and evaluating clinical reasoning performance. We first created the ICU-REACT seed dataset using LLM-assisted generation, followed by structured review and refinement by 19 clinicians to ensure that the final samples reflected realistic ICU decision-making scenarios. We then augmented this seed set to create the training sets, fine-tuned Llama [28], Gemma [29], and Baichuan [18] backbones using low-rank adaptation (LoRA) supervised fine-tuning (SFT) to obtain Clin-REACT models, and evaluated performance against open-source general-purpose and medical LLMs on internal and external benchmarks.

### 2.1 ICU-REACT captures multidimensional clinical reasoning, enabling reasoning-focused training

We created ICU-REACT, a decision-focused clinical reasoning and information retrieval dataset for the ICU, developed through a clinician-in-the-loop annotation framework for fine-tuning and benchmarking LLMs. Specifically, the seed set was

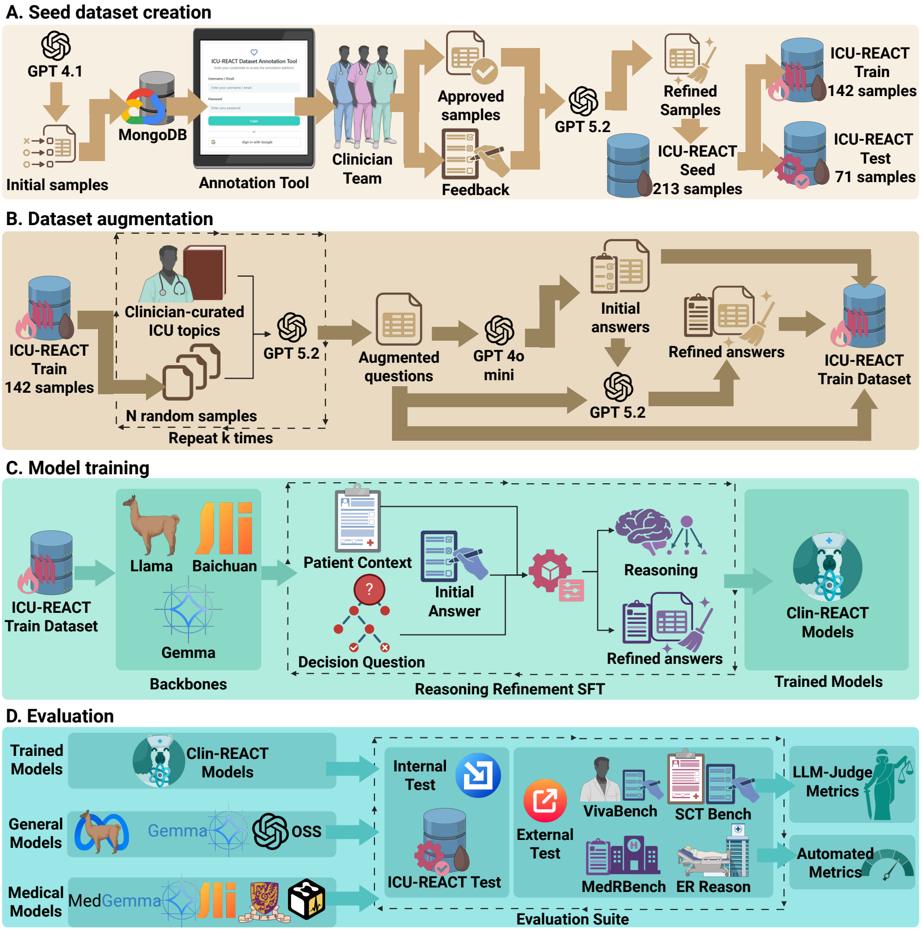

**Fig. 1** Workflow overview of the ICU-REACT and Clin-REACT pipeline. (A) Seed dataset creation: GPT-4.1, selected for its state-of-the-art instruction-following performance at the time of initial generation, produces candidate samples that are stored in MongoDB and reviewed by clinicians through an online annotation tool. Approved samples are then refined with GPT-5.2, the state-of-the-art model available at the time of the experiments, using clinicians' written feedback to produce the ICU-REACT seed set ($n = 213$). (B) Dataset augmentation: the seed set is divided into a training seed ($n = 142$) and held-out test set ($n = 71$). Clinician-curated ICU topics and $n$ randomly sampled training examples are used as few-shot context for GPT-5.2 to generate new questions. GPT-4o-mini is intentionally used to produce imperfect initial answers requiring refinement, after which GPT-5.2 generates improved answers using the question and initial response. This process is repeated for $k$ rounds until the target training-set size is reached. (C) Model training: Llama 3.1 8B Instruct, Baichuan M1 14B Instruct, Gemma 4 31B, and Llama 3.3 70B Instruct are fine-tuned through a single supervised fine-tuning stage to generate refined answers and corresponding reasoning from the patient context, decision question, and initial answer. ICU-REACT-Train-Small contained 10,000 samples and was used for the 8B model; ICU-REACT-Train-Medium contained 5,307 hard reasoning-refinement samples and was used for the 14B and 31B models; and ICU-REACT-Train-Large contained 27,973 reasoning-refinement samples and was used for the 70B model. Fine-tuning produced the Clin-REACT 8B, 14B, 31B, and 70B models. (D) Evaluation: Clin-REACT models are compared with open-source general-purpose and medical LLMs on the held-out ICU-REACT test set and four external benchmarks (SCT-Bench, ER-Reason, MedRBench, and VivaBench) using automated and LLM-judge metrics.

curated through LLM-assisted generation and subsequently reviewed and refined by 19 clinicians across multiple specialties, resulting in 213 final samples, a size that is consistent with previous literature [26, 27]. The seed set was then split into training ($n = 142$) and held-out test ($n = 71$) sets. The training seed was augmented via a self-instruct pipeline into three variant-specific training sets: ICU-REACT-Train-Small (10,000 samples to train Clin-REACT 8B), Medium (5,307 hard reasoning-refinement samples to train Clin-REACT 14B and 31B), and Large (27,973 reasoning-refinement samples to train Clin-REACT 70B). The Small, Medium, and Large labels refer to the size of the Clin-REACT models they were designed to train, rather than the size of the training datasets. Each dataset was constructed to balance training-data scale and reasoning complexity with the capacity of the corresponding model architecture and size. Full composition and topic distributions for the training sets are provided in Supplementary Fig. S2.

For benchmarking, ICU-REACT-Test (the held-out test set) comprised 71 clinician-validated questions spanning nine critical-care topics, with greater representation of common ICU problems such as respiratory failure ($n = 16$), hemodynamic instability/shock ($n = 11$), and renal failure/electrolyte disorders ($n = 9$). Rather than testing isolated facts or individual EHR variables, questions were designed to require integration of multiple aspects of the patient record that clinicians routinely consider together when making decisions. A typical question required information from 4 clinical data categories (IQR 3-5), 7 more specific data sub-domains (IQR 5-10), and 18 individual patient variables (IQR 13–26.5). These commonly included vital signs, laboratory results, medications, and other physiologic measurements, reflecting the multidimensional information routinely synthesized during ICU care. Detailed test-set composition, topic distributions, and data-category breakdowns are provided in Supplementary Fig. S3.

### 2.2 Clin-REACT training transfers across diverse clinical reasoning benchmarks

Clin-REACT training improved performance beyond the in-domain ICU-REACT tasks, with Clin-REACT models generally performing better across four external clinical-reasoning benchmarks with different task formats and evaluation criteria (Fig. 2). Clin-REACT 31B achieved the highest macro score across all five benchmarks at 50.4 (±15.5 SD), compared with 48.5 (±15.8) for GPT-OSS 120B and 48.3 (±17.5) for Gemma 4 31B. As expected, the largest gains were seen on ICU-REACT, where the three best-performing models were Clin-REACT variants. Clin-REACT 70B achieved the highest score at 45.0 (95% CI: 42.8-47.4), compared with 40.6 (38.1-43.0) for the strongest baseline.

Importantly, these gains carried over to benchmarks that differed from ICU-REACT in clinical setting, task structure, and scoring methodology. Clin-REACT models achieved the highest overall scores on three of the four external benchmarks, with the best-performing variants reaching 51.4 (46.5-55.8) on ER-Reason, 47.9 (47.0-48.7) on MedRBench, and 33.5 (32.2-34.8) on VivaBench. They also remained competitive on SCT-Bench: Clin-REACT 31B scored 75.5 (69.8-81.0), compared with 77.6 (72.1-83.1) for the top-performing model, while Clin-REACT 70B outperformed

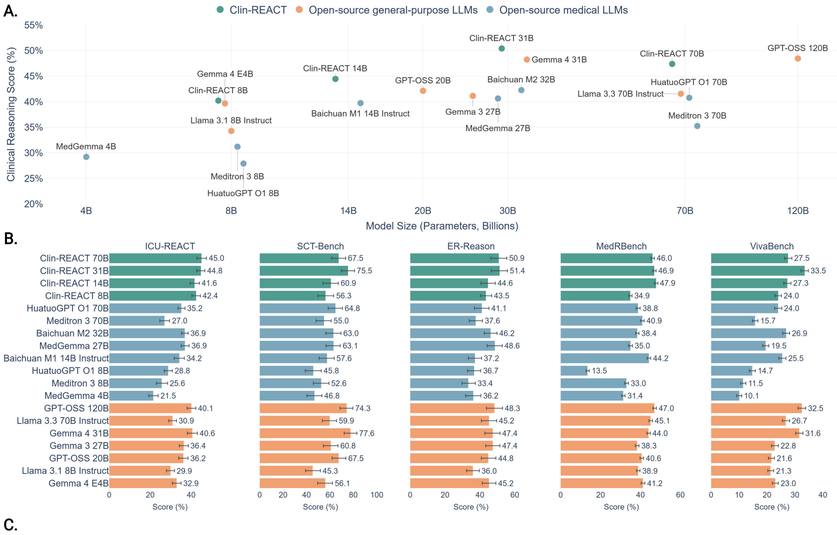

**Fig. 2** Overall performance. (A) Relationship between model size and macro-average performance across all five clinical reasoning benchmarks evaluated (ICU-REACT, SCT-Bench, ER-Reason, MedRBench, and VivaBench). Models are colored by group: Clin-REACT models, open-source general-purpose LLMs, and open-source medical LLMs. (B) Benchmark-level performance of all models on ICU-REACT, SCT-Bench, ER-Reason, MedRBench, and VivaBench. Bars denote mean scores across each benchmark's metrics, and error bars represent 95% confidence intervals. (C) Metric-level heatmap summarizing performance across the individual evaluation dimensions within each benchmark. Models are ordered by group and performance is displayed as percentage scores.

the strongest medical model (67.5 vs. 64.8). Together, these results suggest that the skills learned during Clin-REACT training extended beyond the source benchmark to a broader range of clinical-reasoning tasks.

Metric-level results showed a similar pattern. On ICU-REACT, Clin-REACT models showed their largest gains in information retrieval and reasoning, including the highest parent-variable F1 (45.1, 95% CI: 42.0-48.3) and reasoning score (60.9, 58.2-63.4). On the external benchmarks, these gains appeared in different ways, including better identification of decision factors, differential reasoning, and treatment planning on ER-Reason; stronger assessment recommendation recall and diagnosis and treatment accuracy on MedRBench; and higher key-information recall and final-diagnosis accuracy on VivaBench. Thus, improvements in clinically grounded retrieval and reasoning transferred to tasks requiring different combinations of information selection, diagnosis, and treatment planning.

When Clin-REACT models did not achieve the highest score on a given metric, the differences often reflected precision-recall tradeoffs rather than a clear loss of capability. For example, on MedRBench, Clin-REACT 31B had lower assessment-recommendation recall than GPT-OSS 120B (48.0 vs. 54.7) but higher precision (29.8 vs. 23.0), while achieving similar diagnosis accuracy (66.0 vs. 67.1). A similar pattern appeared on VivaBench, where Clin-REACT 31B showed higher key-information precision but lower recall than GPT-OSS 120B. Overall, the consistency of these results across diverse external benchmarks suggests that Clin-REACT training yielded transferable clinical-reasoning skills rather than simply improving performance on ICU-REACT. Detailed model- and metric-level comparisons with confidence intervals and statistical testing are provided in Supplemental Section S4, with comparisons against four proprietary frontier LLMs reported in Supplemental Section S6.

### 2.3 Fine-tuning consistently improves performance over backbone models across clinical reasoning benchmarks

Pairwise comparisons with 14 baseline models across five clinical-reasoning benchmarks showed that Clin-REACT training consistently improved the performance of each backbone model (Fig. 3). Across model sizes, Clin-REACT variants achieved more significant wins and fewer significant losses than their corresponding backbones, with improvements extending beyond ICU-REACT to external reasoning tasks.

The gains were especially clear for the 8B and 70B models. Clin-REACT 8B increased from 12 significant wins with the Llama 3.1 8B Instruct backbone to 29, while reducing significant losses from 43 to 22. The largest improvement was on ICU-REACT (+9 wins), but gains were also seen on ER-Reason (+5), SCT-Bench (+2), and VivaBench (+2). Similarly, Clin-REACT 70B increased from 34 to 59 significant wins and reduced losses from 16 to 5 relative to Llama 3.3 70B Instruct. These improvements were seen across all five benchmarks, including substantial gains on ER-Reason (+7) and SCT-Bench (+4).

Clin-REACT training also strengthened the already competitive 14B and 31B backbones. Clin-REACT 14B increased significant wins from 32 to 44 and reduced losses from 21 to 6, with improvements extending beyond ICU-REACT to ER-Reason, SCT-Bench, and MedRBench. Clin-REACT 31B showed the strongest comparison profile, with 67 significant wins and no significant losses across 70 comparisons. Although its Gemma 4 31B backbone was already highly competitive, with 58 wins and only two losses, Clin-REACT training added nine wins and dropped both losses, with most of the additional gains occurring on ER-Reason and MedRBench.

Overall, these pairwise comparisons show that Clin-REACT training improved the relative performance of every backbone across a diverse set of clinical-reasoning tasks. The consistency of these improvements across model sizes and external benchmarks provides further evidence that the reasoning and information-retrieval skills learned

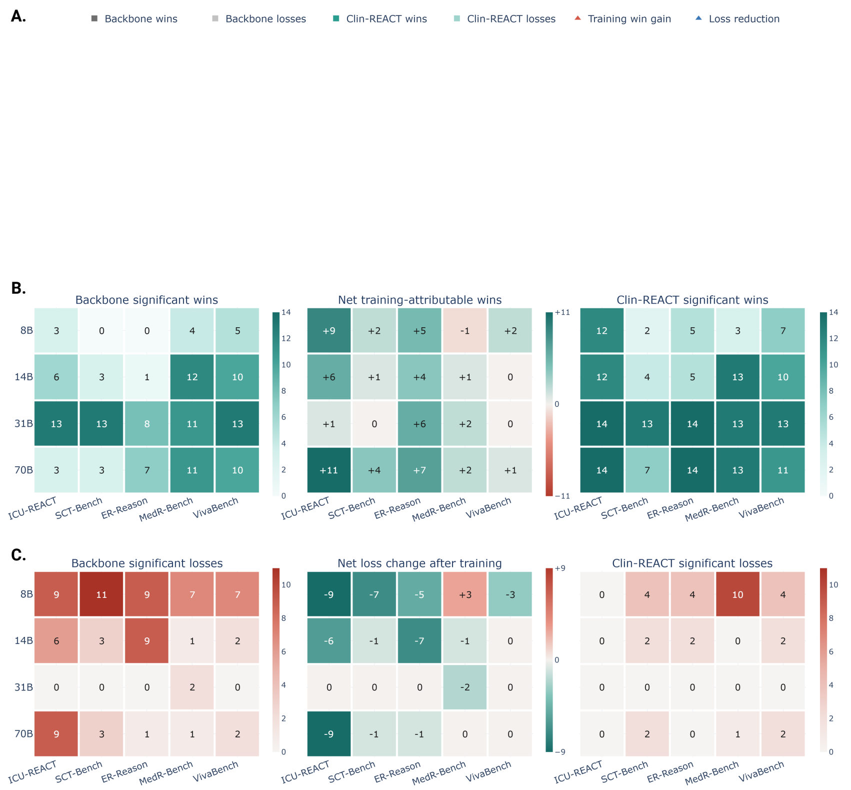

**Fig. 3** Clin-REACT performance gains. Each Clin-REACT model is compared against its untrained backbone (8B, 14B, 31B, and 70B) across five benchmarks. A ”win” is a pairwise comparison in which a model significantly outperforms a competing model, and a ”loss” is one in which it is significantly outperformed; significance was determined using two-sided Wilcoxon signed-rank tests ($p < 0.05$). Comparing a backbone's win/loss counts against its corresponding fine-tuned Clin-REACT model isolates the effect of ICU-REACT training. (A) Aggregate counts of significant wins (bars above the axis) and losses (bars below the axis) summed across all benchmark comparisons, shown for each backbone (gray) and its corresponding Clin-REACT model (teal). The red arrow marks the increase in wins and the blue arrow marks the decrease in losses attributable to training. Across all four scales, Clin-REACT increases wins and reduces losses. (B) Significant wins broken down by benchmark: backbone wins (left), Clin-REACT wins (right), and the net difference between them (middle). In the middle heatmap, positive values indicate additional wins gained through training and negative values indicate wins lost. (C) Significant losses broken down by benchmark: backbone losses (left), Clin-REACT losses (right), and the net change (middle). In the middle heatmap, negative values indicate fewer losses after training (an improvement in performance) and positive values indicate more losses (a decrease in performance).

during training transferred beyond the ICU setting. Heatmaps showing the magnitude of differences between each Clin-REACT variant and all baseline models, including gains over their corresponding backbones, are provided in Supplemental Section S4.

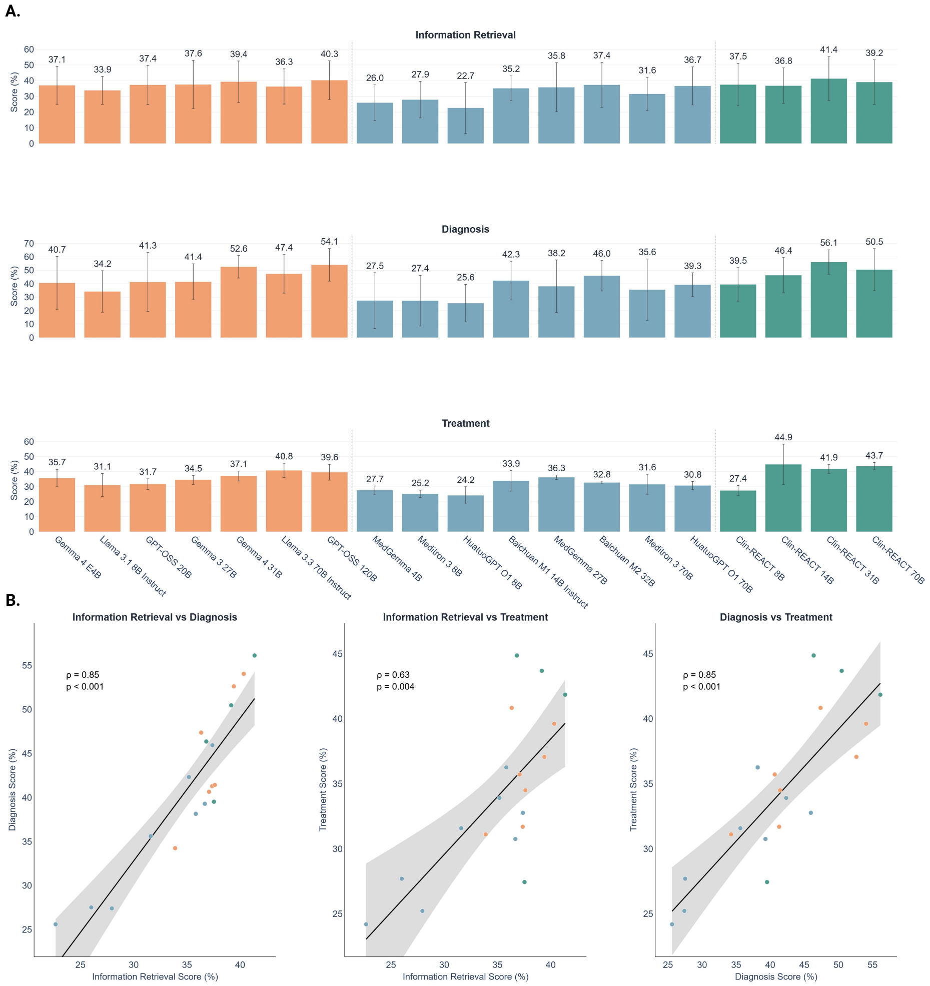

**Fig. 4** Performance across different task types. (A) Task-level scores for information retrieval, diagnosis, and treatment across Clin-REACT models, open-source general-purpose LLMs, and open-source medical LLMs. Task scores were calculated by aggregating relevant benchmark metrics within each task domain. Bars denote mean performance across included metrics, and error bars denote the standard deviation across metrics. (B) Pairwise associations between model performance across information retrieval, diagnosis, and treatment categories. Points represent individual models, colored by model group, and fitted lines show the estimated linear relationships with shaded 95% confidence intervals. Associations were evaluated using Spearman rank correlation tests, with correlation coefficients and corresponding p-values shown in each panel.

### 2.4 Performance gains extend from information retrieval reasoning to downstream diagnosis and treatment

To assess whether Clin-REACT performance generalized across different aspects of clinical reasoning, benchmark-specific metrics were grouped into three task categories: information retrieval, diagnosis, and treatment (Fig. 4A). Information retrieval included ICU-REACT parent variable F1 and variable F1 scores, ER-Reason decision factors accuracy, MedRBench assessment recommendation precision and recall, and VivaBench overall key-information precision and recall. Diagnosis included ER-Reason differential accuracy, MedRBench diagnosis accuracy, and VivaBench final diagnosis accuracy. Treatment included ER-Reason treatment planning accuracy and MedRBench treatment accuracy.

Clin-REACT models performed strongly across all three task categories, rather than showing gains only in information retrieval, the most emphasized task during training. Clin-REACT 31B achieved the highest overall scores for information retrieval and diagnosis, at 41.4 (SD 14.0) and 56.1 (SD 9.1), respectively, while Clin-REACT 14B achieved the highest treatment score at 44.9 (SD 13.4). Clin-REACT 70B was also competitive across all three categories. Together, these results show that Clin-REACT variants matched or outperformed the strongest general-purpose and medical baselines across information retrieval, diagnosis, and treatment, suggesting that the skills learned during training extended to multiple stages of clinical reasoning.

Comparisons with the corresponding backbone models showed that improvements on these tasks was attributable to Clin-REACT training. Clin-REACT 31B and 70B improved over Gemma 4 31B and Llama 3.3 70B Instruct, respectively, across all three categories. Clin-REACT 14B also improved in retrieval and diagnosis and showed a particularly large gain in treatment performance relative to Baichuan M1 14B Instruct (44.9 vs. 33.9). Clin-REACT 8B improved in retrieval and diagnosis compared with Llama 3.1 8B Instruct, although treatment performance had a slight decrease (27.4 vs. 31.1). Overall, these findings suggest that the benefits of Clin-REACT training generally extended beyond information retrieval to downstream diagnosis and treatment tasks.

Performance across the three task categories was also positively associated across models (Fig. 4B). Information-retrieval and diagnosis scores were strongly correlated ($\rho = 0.85$, $p < 0.001$), as were diagnosis and treatment scores ($\rho = 0.85$, $p < 0.001$); information retrieval was moderately correlated with treatment performance ($\rho = 0.63$, $p = 0.004$). These associations indicate that models that were better at identifying clinically relevant information also tended to perform better on downstream diagnosis and treatment tasks, highlighting information selection as an important step of clinical reasoning.

### 2.5 Performance improvements are consistent across diverse clinical content

Performance across the nine ICU topic categories showed that Clin-REACT improvements were spread across a broad range of clinical problems rather than driven by only a few conditions (Fig. 5). The clearest pattern was in reasoning quality, where a Clin-REACT variant achieved the highest score in every ICU topic. These gains were seen across major critical-care areas, including sepsis and severe infections, hemodynamic instability and shock, respiratory failure, renal and electrolyte disorders, cardiac emergencies, hematologic and coagulation disorders, and sedation, pain, and delirium management. For example, Clin-REACT achieved reasoning scores of 58 versus 40 for the strongest baseline in sepsis and severe infections, 60 versus 44 in hemodynamic

A.

B.

C.

**Fig. 5** Performance across topics in ICU-REACT test set. Heatmaps showing model performance across the nine topic areas in ICU-REACT test set for (A) parent variable F1, (B) variable F1, and (C) reasoning score. Rows denote ICU topics and columns denote evaluated models, ordered from left to right with open-source general-purpose LLMs followed by open-source medical LLMs and Clin-REACT models. Cell values represent percentage scores, with warmer colors indicating higher performance.

instability and shock, 59 versus 45 in respiratory failure, and 67 versus 48 in renal failure and electrolyte disorders.

Information-retrieval performance varied more across topics but remained competitive with the strongest baselines. Clin-REACT models achieved the highest variable-level retrieval scores in several high-acuity areas, including respiratory failure and renal failure and electrolyte disorders, and matched or closely approached the strongest baselines in sepsis and severe infections and hemodynamic instability and shock. Parent-variable retrieval followed a similar pattern, with the best Clin-REACT variants generally within 1-2 points of the top baseline across these major ICU domains.

Overall, these results show that Clin-REACT training improved reasoning across a wide range of ICU presentations while maintaining strong retrieval of clinically relevant information. Similar gains were also seen across related clinical domains in the external benchmarks, further supporting the transfer of these capabilities beyond the ICU setting (Supplemental Section S7).

### 2.6 Training improves scores across evaluation dimensions

To better understand how Clin-REACT training changed the quality of model responses beyond overall benchmark scores, we evaluated five evaluation dimensions on ICU-REACT: reasoning correctness, reasoning synthesis, task faithfulness, safety, and critical-anchor identification (Fig. 6A). Clin-REACT models consistently outperformed general-purpose and medical baselines in both reasoning correctness and synthesis, with correctness scores ranging from 41.0% to 49.4% and synthesis scores from 46.9% to 67.6%. These results suggest that Clin-REACT models were not only more likely to provide clinically appropriate explanations, but also better at bringing relevant evidence together into a coherent rationale.

The clearest advantages were seen in task faithfulness and safety. Clin-REACT models achieved task-faithfulness scores of 96.7%-99.8%, compared with 41.5%-95.1% for general-purpose models and 60.6%-93.0% for medical models. Safety followed a similar pattern, with Clin-REACT scores (78.6%-93.9%) exceeding both general-purpose (34.2%-76.1%) and medical (51.6%-67.1%) baselines.

Identifying critical anchors remained the most difficult dimension for all models, although Clin-REACT still achieved the strongest overall performance (22.4%-34.0%). Overall, these findings show that Clin-REACT training improved not only the correctness and coherence of clinical reasoning, but also how well models followed the intended task and avoided unsafe responses. At the same time, reliably identifying the most clinically important evidence remains an important area for improvement.

### 2.7 Information retrieval scores improve across multiple clinically relevant data categories

Information-retrieval performance differed across clinical data types, but Clin-REACT models showed clear strengths in retrieving laboratory measurements, structured scores and assessments, and imaging findings (Fig. 6B). A Clin-REACT variant achieved the highest F1 score among all evaluated models in each of these categories, reaching 38.9% for laboratory measurements, 38.1% for scores and assessments, and 26.6% for imaging. Clin-REACT models also remained competitive for physiology and medications, nearly matching the strongest general-purpose model for physiology (45.9% vs. 46.0%) and outperforming the strongest medical baseline for medication retrieval.

Performance was more variable for diagnosis, intervention, and administrative information. Clin-REACT models remained competitive with medical baselines for

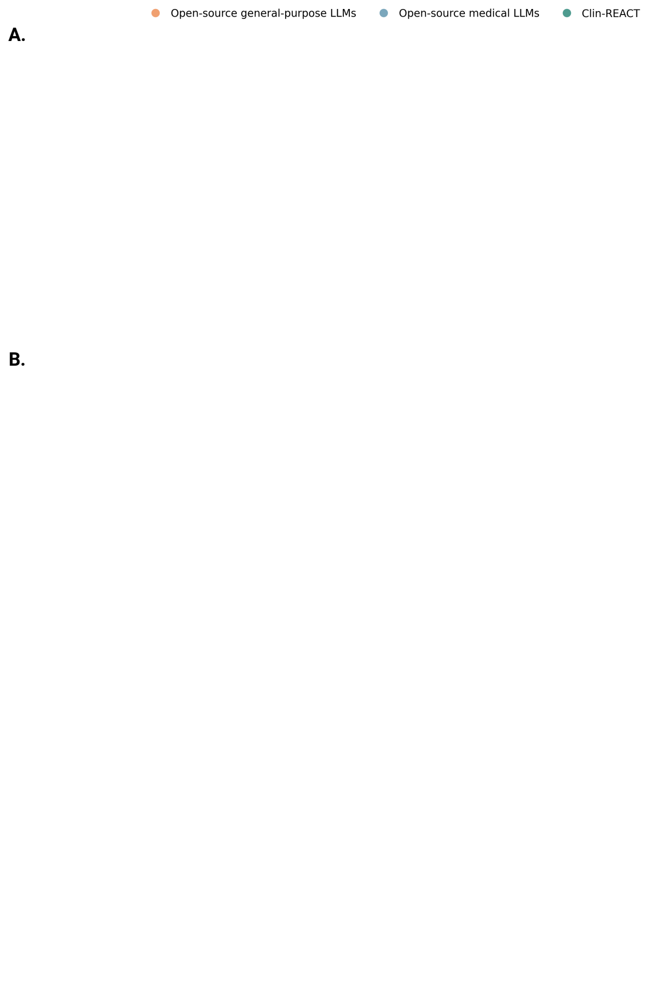

**Fig. 6** Performance across rubric dimensions and clinical data categories. (A) Cleveland dot plots summarizing model performance across the five ICU-REACT rubric dimensions: reasoning correctness, reasoning synthesis, task faithfulness, safety, and critical anchor. (B) Cleveland dot plots showing variable-level F1 performance stratified by the clinical data categories represented in ICU-REACT questions, including physiology, laboratory measurements, medications, diagnoses, interventions, scores and assessments, imaging, and administrative information. Each dot represents the score for a given model on a specific dimension or category. Models are grouped by open-source general-purpose LLMs, open-source medical LLMs, and Clin-REACT models.

intervention retrieval but had lower performance compared to the strongest general-purpose model, while diagnosis retrieval was modestly lower than the best general-purpose and medical baselines. Administrative information was challenging for all models and had the lowest retrieval scores overall. These results show that Clin-REACT training improved retrieval across several clinically important data types, while also highlighting diagnosis, intervention, and administrative information as areas for further improvement. Similar category-level gains were seen on external benchmarks, suggesting that these retrieval skills extended beyond the ICU-REACT test set (Supplemental Section S8).

#### 2.8 ICU-REACT performance aligns with external clinical reasoning benchmarks

To assess concordance between ICU-REACT and external measures of clinical reasoning, we calculated model-level Spearman rank correlations between ICU-REACT performance and scores on SCT-Bench, ER-Reason, MedRBench, and VivaBench (Fig. 7). ICU-REACT showed strong positive correlations with SCT-Bench ($\rho = 0.76$, ($p < 0.001$)), ER-Reason ($\rho = 0.78$, ($p < 0.001$)), and VivaBench ($\rho = 0.80$, ($p < 0.001$)), and a more moderate but significant correlation with MedRBench ($\rho = 0.51$, ($p = 0.025$)). Models that performed well on ICU-REACT also tended to perform well on external clinical-reasoning benchmarks, suggesting that ICU-REACT captures skills that are relevant to broader clinical reasoning.

Metric-level correlations showed a similar pattern across reasoning dimensions (Fig. 7B). ICU-REACT retrieval and reasoning metrics were strongly correlated to one another and were positively associated with external measures of decision-factor identification, differential diagnosis, treatment planning, and final diagnosis. The strongest cross-benchmark relationships were seen with SCT-Bench, ER-Reason decision-factor performance, and VivaBench final diagnosis accuracy, while diagnosis and treatment metrics were also closely aligned across MedRBench and VivaBench. In contrast, precision metrics showed weaker or occasionally inverse relationships with recall measures, reflecting differences in retrieval behavior and precision-recall tradeoffs across models. Overall, these correlations suggest that ICU-REACT measures reasoning abilities that overlap with established clinical-reasoning benchmarks.

### 3 Discussion

In this study, we developed ICU-REACT, a decision-focused dataset for evaluating information retrieval and clinical reasoning in critical care, and Clin-REACT, a suite of fine-tuned LLMs optimized for information retrieval and reasoning in the ICU. The ICU-REACT dataset was designed to improve clinical reasoning of LLMs and address an important challenge in evaluating LLMs for critical care: assessing whether models can identify relevant patient information under different decision scenarios and use it to generate clinically coherent, task-aligned, and safe reasoning in complex ICU scenarios [30, 31]. Our results demonstrate that reasoning-refinement training on a domain-constrained dataset can yield broad, transferable improvements in clinically relevant reasoning and information-selection behavior that extend well beyond the ICU setting, while surpassing strong open-source general-purpose and medical LLMs.

A central finding of this study is that Clin-REACT models improved clinical reasoning performance not only on in-domain ICU tasks but also across external benchmarks that were not part of training. Although ICU-REACT is constrained to critical-care scenarios, Clin-REACT models achieved leading or highly competitive performance on ER-Reason, a related critical-care benchmark, as well as on

A.

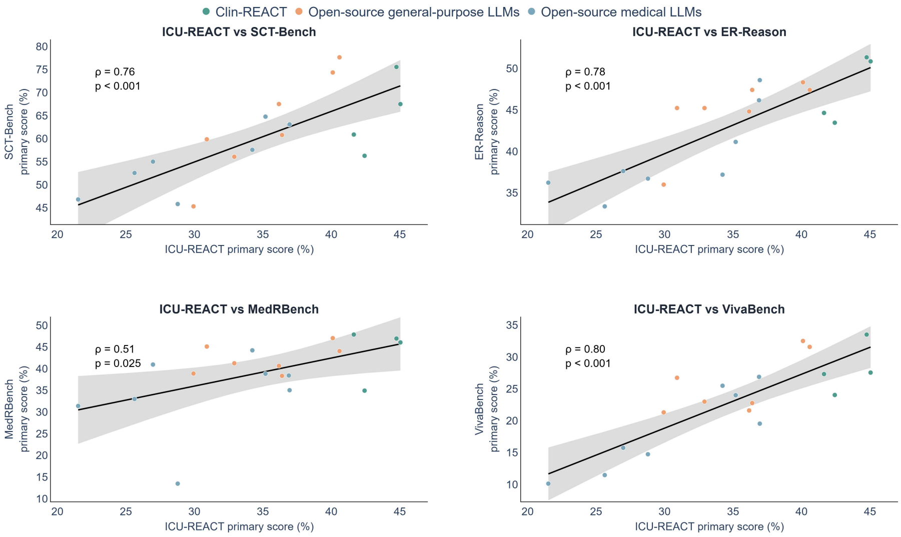

B.

**Fig. 7** Performance correlations between clinical reasoning benchmarks. (A) Associations between models' ICU-REACT average scores and their average scores on SCT-Bench, ER-Reason, MedR-Bench, and VivaBench. Points represent individual models and are colored by model group, with solid lines indicating fitted linear trends with shaded 95% confidence intervals. Associations were evaluated using Spearman rank correlation tests, with correlation coefficients and corresponding p-values shown in each panel. (B) Heatmap of pairwise Spearman rank correlations among individual metrics across the five clinical reasoning benchmarks evaluated. Cell values denote correlation coefficients, with positive and negative associations represented by teal and red shading, respectively.

more general clinical reasoning benchmarks such as SCT-Bench, MedRBench, and VivaBench. For instance, Clin-REACT 31B achieved the highest overall macro score across all benchmarks (50.4 ± 15.5 SD) and the best performance on ER-Reason (51.4) and VivaBench (33.5), while remaining competitive on SCT-Bench and MedRBench (Fig. 2). Importantly, these improvements were not limited to tasks that closely resembled ICU-REACT or to a single type of evaluation. The external benchmarks tested different aspects of clinical reasoning, including decision-making under uncertainty, diagnosis and treatment selection, information seeking, and concept-level reasoning, using scoring approaches ranging from agreement with expert response distributions to retrieval and accuracy deterministic metrics. The fact that Clin-REACT improved across these diverse tasks and evaluation methods makes it less likely that the gains were driven only by memorization, dataset-specific response patterns, or alignment with an LLM evaluator. Instead, the results suggest that the reasoning skills learned from ICU-REACT transferred beyond the training setting, improving clinically relevant reasoning and information selection across different clinical contexts and task formats.

Clin-REACT training produced consistent improvements over each model's respective backbone across model families and scales. Relative to their backbones, Clin-REACT variants gained net statistically significant wins on our pairwise benchmark comparisons at every scale, ranging from 9 net wins for Clin-REACT 31B over its already strong Gemma 4 31B backbone to 25 net wins for Clin-REACT 70B over Llama 3.3 70B Instruct (Fig. 3). Notably, Clin-REACT 31B achieved 67 significant wins with no significant losses across 70 comparisons, and removed both of the losses observed for its backbone. The gains on SCT-Bench provide a particularly clear example of this transfer. Rather than simply selecting a diagnosis or semantically matching an expected response, SCT-Bench asks models to quantify how new clinical information changes the likelihood of a diagnostic hypothesis under uncertainty. Despite this task formulation not being explicitly represented in ICU-REACT training, Clin-REACT improved over its respective backbone by +11.0% for the 8B model ($p < 0.01$), +3.3% for the 14B model ($p > 0.05$), and +7.6% for the 70B model ($p < 0.01$), showing better agreement with clinician response distributions and providing evidence that the learned reasoning behaviors generalized beyond the structure of the training task itself. These improvements were seen across different model families (Llama, Gemma, and Baichuan) indicating that the benefits of ICU-REACT training were not specific to a single architecture. The magnitude of the gains, however, varied across models. Stronger backbones, such as Gemma 4 31B, showed smaller improvements, given they already performed well before fine-tuning. On the other hand, models with more room to improve showed larger gains. Importantly, our data contamination analyses found no evidence of substantial overlap or benchmark leakage between ICU-REACT training data and the external benchmarks that could readily explain these results (Supplemental Section S10), supporting the interpretation that the improvements reflect transferable learning rather than memorization.

Notably, these gains were achieved through lightweight low-rank adaptation (LoRA) fine-tuning rather than extensive retraining. Clin-REACT models were therefore highly parameter-efficient, with smaller variants often matching or outperforming much larger general-purpose and medical LLMs. For example, Clin-REACT 14B achieved higher overall clinical reasoning performance than MedGemma 27B and Baichuan M2 32B, while Clin-REACT 8B exceeded larger medical models such as Baichuan M1 14B Instruct and Meditron 3 70B. This pattern also held at larger model sizes. Clin-REACT 31B achieved the highest overall performance, outperforming HuatuoGPT O1 70B and GPT-OSS 120B despite having roughly one quarter as many parameters as the latter. Clin-REACT 70B also clearly outperformed HuatuoGPT O1 70B and exceeded GPT-OSS 120B on ICU-REACT and ER-Reason. The fact that relatively lightweight fine-tuning could produce models that matched or surpassed much larger and more heavily trained systems suggests that the gains came from the quality of ICU-REACT and the reasoning-refinement training approach, rather than model size or training scale alone.

Beyond information retrieval, Clin-REACT models also improved on downstream diagnosis and treatment tasks. In our task-level analysis, Clin-REACT models achieved the strongest or highly competitive performance across all three categories. Clin-REACT 31B had the highest scores for information retrieval (41.4) and diagnosis (56.1), while Clin-REACT 14B achieved the highest treatment score (44.9) (Fig. 4A). Relative to their backbones, Clin-REACT models improved information retrieval and diagnostic performance at all four scales. This pattern is reinforced by the moderate-to-strong correlations we observed among task categories (information retrieval and diagnosis, $\rho = 0.85$; diagnosis and treatment, $\rho = 0.85$; information retrieval and treatment, $\rho = 0.63$) (Fig. 4B). One possible explanation for these gains is that better identification and prioritization of clinically relevant information gives models stronger evidence for subsequent diagnostic and treatment decisions. This may help explain why Clin-REACT training improved not only information retrieval, but also diagnostic performance on external benchmarks such as MedRBench and VivaBench. Models that more effectively recognize which findings are relevant may also be better positioned to reach the correct diagnosis or treatment decision. Although these associations do not establish that improved retrieval directly causes better downstream performance, they suggest that the benefits of Clin-REACT training extend across multiple stages of clinical decision-making rather than being limited to information extraction alone.

The reasoning-refinement approach seemed to play an important role in these results. Among the training tasks we tested, asking models to improve a flawed initial response had the best results, outperforming alternatives such as variable selection and context or question generation (see ablations in Supplemental Section S5). One possible explanation is that models learn more from correcting imperfect reasoning than from simply imitating correct answers, since the refinement process exposes them to failure modes and the steps needed to fix them [32, 33]. Our t-SNE analyses support this interpretation, showing greater diversity among refined reasoning texts than initial reasoning texts (Supplementary Fig. S2), suggesting that refinement provides a richer training signal. More broadly, these findings support a framework in which clinician-curated reasoning serves as the foundation, LLM-based augmentation amplifies that signal, and fine-tuning teaches models reasoning and information-selection skills that transfer across clinical tasks.

Our analyses also revealed meaningful limitations of existing LLMs on ICU clinical reasoning that ICU-REACT was able to surface. Across ICU topics and rubric dimensions, general-purpose and medical models frequently struggled, whereas Clin-REACT models showed consistent improvement. A Clin-REACT variant achieved the highest reasoning score across all nine ICU topic categories, with pronounced advantages in critical domains such as sepsis, hemodynamic instability/shock, respiratory failure, and renal failure (Fig. 5). The evaluation-dimension analysis was especially informative in areas where many models still struggle. For safety, general-purpose models scored 34.2-76.1 and medical models 51.6-67.1, showing that even strong baselines sometimes produced recommendations or overlooked considerations that could raise safety concerns. In contrast, Clin-REACT models performed better (78.6-93.9) (Fig. 6A). Critical-anchor identification was even more challenging. This metric measured whether models recognized the most important clinical evidence supporting a decision. General-purpose models scored only 6.4-22.4 and medical models 7.7-17.9, suggesting that many models failed to center their reasoning on the most relevant findings. Clin-REACT achieved the strongest performance (22.4-34.0), but scores remained relatively low across all models, highlighting reliable identification of the most consequential clinical evidence as an important remaining challenge.

Clin-REACT models similarly improved on reasoning correctness, reasoning synthesis, and task faithfulness, and demonstrated more comprehensive information retrieval across clinical data categories, achieving the strongest F1 in three of eight categories, including laboratory measurements, scores and assessments, and imaging, and remaining competitive in other categories (Fig. 6B). Notably, general-purpose and medical models tended to rely more heavily on routinely documented data, such as laboratory results, physiology, and medications, while more often missing clinically interpretive information such as scores/assessments, and imaging. Clin-REACT training helped shift attention toward these data types. Clin-REACT 31B achieved the highest F1 for scores and assessments (38.1), while Clin-REACT 70B performed best for imaging (26.6). This suggests that training with ICU-REACT encouraged models to consider a broader and more clinically complete set of evidence, rather than defaulting to the information that is most readily available.

A notable observation is that open-source medical LLMs generally underperformed on these clinical reasoning benchmarks, often having lower performance than general-purpose models of comparable or smaller size. This finding is consistent with recent results comparing general-purpose and specialized clinical models [34]. Because many medical LLMs are optimized on multiple-choice question-answering benchmarks, this pattern raises the possibility that such training incentivizes pattern recognition and answer memorization rather than the generative, multi-step reasoning required for realistic clinical decision-making [35, 36]. Our analysis comparing performance across medical multiple-choice and clinical-reasoning benchmarks further supports this interpretation, showing that stronger multiple-choice performance did not consistently correspond to stronger performance on clinically grounded reasoning tasks (see Supplemental Section S9). If so, strong performance on multiple-choice medical benchmarks may not fully reflect a model's clinical reasoning ability. Open-ended frameworks such as ICU-REACT, which require models to identify relevant information and reason through it, may provide a more realistic assessment of models intended for clinical use.

Finally, our correlation analyses support ICU-REACT's validity as a clinical reasoning benchmark. Model-level ICU-REACT performance correlated moderately to strongly with SCT-Bench ($\rho = 0.76$), ER-Reason ($\rho = 0.78$), and VivaBench ($\rho = 0.80$), and more moderately but still significantly with MedRBench ($\rho = 0.51$) (Fig. 7A). These relationships were also seen at the metric level, with ICU-REACT metrics being closely correlated with decision-factor identification, diagnosis, and recall metrics across the external benchmarks. More broadly, the correlation patterns across metrics and datasets showed that models were ranked similarly across different evaluations (Fig. 7B). Together, these findings suggest that ICU-REACT captures a meaningful signal of clinical reasoning ability that is consistent with established benchmarks, while also testing ICU-specific skills that other benchmarks do not directly assess. The weaker or occasionally negative correlations among precision metrics also highlight an important precision-recall trade-off in clinical reasoning, which a decision-focused benchmark such as ICU-REACT may help reveal.

Our study has several limitations. First, our evaluation relied on automated metrics and LLM-as-judge evaluations rather than direct expert review of Clin-REACT outputs. Although LLM-based evaluation can scale across many models and has shown reasonable agreement with human raters, it cannot fully replace clinician judgment of response quality, safety, and clinical appropriateness. A related concern is that GPT-5.2 was used both to generate training dataset and to evaluate selected endpoints, including ICU-REACT reasoning. This teacher-judge overlap could favor responses that resemble the model's preferred reasoning style. However, this limitation does not apply equally across all of our evaluations. Clin-REACT also improved on independently scored outcomes, including agreement with human expert response distributions on SCT-Bench and deterministic measures of diagnosis, treatment, and information retrieval on MedRBench, VivaBench, and ICU-REACT. In addition, ICU-REACT reasoning was scored using rubrics derived from clinician ground-truth responses. The consistency of the results across these different evaluation approaches makes evaluator preference alone unlikely to explain the overall gains, although prospective evaluation by practicing clinicians will be essential.

Second, the ICU-REACT test set is relatively small, with 71 questions. Although this is similar in scale to related benchmarks such as ER-Reason and covers nine common critical-care topics, it cannot represent the full range of cases and edge conditions encountered in ICU practice. ICU-REACT should therefore be viewed as a measure of reasoning across representative ICU scenarios rather than a comprehensive assessment of critical-care reasoning. Third, Clin-REACT models were trained using LoRA fine-tuning alone. This lightweight approach was effective and allowed smaller models to compete with or outperform much larger models. However, we did not explore more advanced approaches such as reinforcement learning from verifiable rewards or preference-based optimization methods such as Group Relative Policy Optimization (GRPO). These strategies may provide additional improvements in reasoning quality. Fourth, although the seed examples used to build ICU-REACT were reviewed by clinicians, the augmented training corpus itself was not clinically validated. The synthetic data may therefore contain factual errors, stylistic patterns, or biases inherited from the teacher model. This limits how strongly the augmented corpus can be described as clinically validated. Nevertheless, the consistent gains across independently developed external benchmarks and different evaluation methods suggest that the training data captured useful and transferable reasoning signals rather than simply reinforcing patterns specific to the synthetic corpus.

Finally, ICU-REACT has limitations related to annotation and clinical representation. Although the dataset was reviewed by clinicians from several specialties, the annotator pool did not include some sub specialties that are highly relevant to ICU care, including neurology, nephrology, and cardiology. This may limit the depth of validation in those areas. In addition, nearly all annotators came from a single institution, with only one clinician from an external site. As a result, some aspects of the dataset may reflect institution-specific practices or conventions, which could limit generalization across health systems.

These limitations point to several directions for future work. One important next step is to integrate Clin-REACT directly with EHR systems using our OMOP-aligned taxonomy of clinical variables, allowing models to retrieve and reason over structured patient data in real time. More advanced training strategies, including reinforcement learning approaches such as GRPO, may further improve reasoning beyond supervised fine-tuning. Prospective evaluation with practicing clinicians will also be critical for assessing safety, usefulness, and performance in realistic clinical settings. Finally, developing ICU-REACT datasets tailored to specific ICU environments, such as medical, surgical, cardiac, or neurological intensive care, could help train models that better reflect the patient populations, workflows, and decision-making needs of each setting.

## 4 Methods

### 4.1 Dataset

We developed ICU-REACT, a clinician-supervised dataset designed to teach LLMs to perform context-aware clinical reasoning and information retrieval. The dataset creation had two main stages: seed dataset creation and dataset augmentation.

#### 4.1.1 Seed dataset creation

We first create the ICU-REACT seed set through LLM-driven generation and clinician expert curation. Specifically, we first employ a multi-agent system responsible for generating diverse, clinically relevant decision-making questions and associated EHR retrieval tasks using controlled prompts and domain ontologies. These initial generations were stored in an online MongoDB and subsequently refined through a structured annotation process conducted by 19 clinicians, including physicians, residents, and medical trainees. Clinicians conducted the review through our online annotation tool to ensure the clinical validity and relevance of the generated data to real-world ICU workflows. All EHR data elements were mapped to a manually crafted subset of standardized concepts from the Observational Medical Outcomes Partnership (OMOP) Common Data Model to facilitate interoperability and reproducibility across EHR systems. The overall process is summarized on Fig. 8.

##### *Initial generation*

The first stage of dataset creation leveraged a multi-agent generation framework consisting of two GPT-4.1-based agents: a Brainstorming Agent and a Reasoning Agent. The GPT-4.1 model was selected for its state-of-the-art instruction-following performance at the time of initial generation.

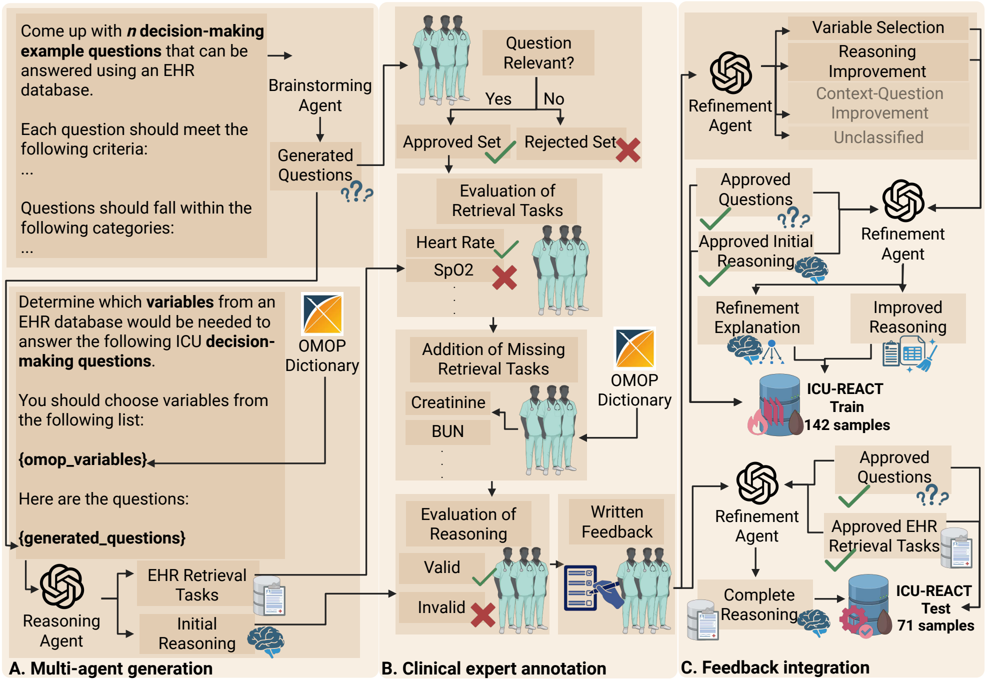

**Fig. 8** Seed dataset creation overview. (A) A brainstorming agent generates candidate ICU decision-making questions that can be answered with data from an EHR database according to predefined clinical criteria and topic categories. A reasoning agent then identifies the EHR retrieval tasks and initial clinical reasoning needed to answer each question, with variables standardized using the OMOP dictionary. (B) Clinical experts review generated questions for relevance, evaluate the completeness and appropriateness of proposed retrieval tasks, add missing variables when necessary, and assess the validity of the initial reasoning while providing written feedback. (C) Written clinician feedback is incorporated by a refinement agent through distinct pipelines for the training and test seed sets. For ICU-REACT-Train, feedback is first categorized as variable-selection, reasoning improvement, context–question improvement, or unclassified. Feedback concerning variable selection and reasoning improvement is then combined with the original patient context–question pair and initial reasoning to generate both an explanation of required revisions and an improved reasoning response. The resulting training samples contain the context, question, initial reasoning, refinement explanation, and improved reasoning. For ICU-REACT-Test, approved questions, retrieval tasks, and written clinician feedback are directly provided to the refinement agent to generate complete reasoning responses incorporating all required EHR variables.

The Brainstorming Agent was prompted to generate $n$ example decision-making questions that could be answered using structured or unstructured EHR data (Fig. 8A). Each question was required to meet specific criteria: it must represent a clinically actionable decision (for example, initiating or adjusting therapy, ordering diagnostics, or assessing patient trajectory), it must be answerable using available EHR data, and it must fall within nine ICU decision domains: Nutrition and Metabolic Support, Sepsis and Severe Infections, Hemodynamic Instability / Shock, Sedation, Pain, and Delirium Management, Respiratory Failure, Neurological Emergencies, Renal

Failure / Electrolyte Disorders, Cardiac Emergencies, Hematologic / Coagulation Issues.

For each generated question, the Reasoning Agent determined which EHR variables would be needed to answer it (Fig. 8A). Variable selection was constrained to a manually crafted subset of standardized terms within the OMOP dictionary (see Supplemental Section S11), ensuring consistency and reproducibility across datasets. The Reasoning Agent produced a structured list of EHR retrieval tasks (for example, heart rate, creatinine, or $SpO_2$) along with a concise reasoning paragraph explaining why those variables were required to address the clinical question. These outputs (decision-making questions, variable retrieval targets, and reasoning explanations) formed the preliminary set of EHR retrieval tasks for expert review.

#### *Clinician annotation*

The second stage involved clinical expert annotation and validation conducted by a team of 19 clinicians across critical care, general surgery, emergency medicine, internal medicine, anesthesiology, and orthopedics. This process ensured the relevance, completeness, and reasoning validity of all generated samples (Fig. 8B).

To facilitate efficient data review and collection, we developed a custom web-based annotation platform (see Supplemental Section S1) that served as the primary interface between clinicians and the data engineering team. The tool provided an interactive environment for reviewing each sample, including the hypothetical patient context, decision-making question, retrieved variables, and model-generated reasoning. Clinicians had the option to approve, reject, or modify each sample, as well as provide written feedback explaining their decisions. All annotations were automatically stored in a centralized MongoDB database on Google Cloud, ensuring version control and traceability.

Clinicians independently reviewed each generated question within the platform to determine its clinical relevance and clarity. To minimize bias, we ensured that each sample was reviewed by two clinicians. Questions that were classified as irrelevant by at least one clinician were excluded, while approved questions proceeded to evaluation of retrieval accuracy. Annotators verified whether the variables identified by the model were relevant for answering each question. Variables that were considered relevant by at least one clinician were kept, while variables that were considered irrelevant by both clinicians were removed. Clinicians then had the option to add missing variables from our manually crafted OMOP dictionary subset to maintain consistent terminology (Fig. 8B).

Following this, clinicians assessed the reasoning explanations provided by the model. Each rationale was evaluated for logical coherence, clinical accuracy, and relevance to the decision-making task. Samples were marked as valid if the reasoning demonstrated appropriate clinical logic supported by the corresponding EHR variables, or invalid if the reasoning was incomplete or clinically inconsistent (Fig. 8B). Approved samples were then split into two sets: an ICU-REACT train seed set for LLM-driven augmentation for model training and an ICU-REACT test set for evaluation of models. This separation ensured that augmented data would not overlap with test cases, mitigating data leakage and helping preserve the integrity of subsequent model assessments. All validation results and written reviewer notes were collected through the annotation platform for subsequent feedback integration for both sets.

***Feedback integration***

To refine approved samples using written clinician feedback, we implemented a feedback integration pipeline leveraging GPT-5.2 as a refinement agent. The GPT-5.2 model was selected for its state-of-the-art status at the time of the experiments. Specifically, we used two different routes for feedback integration on both ICU-REACT seed sets. For the ICU-REACT test set, written feedback was directly inputted into the refinement agent along with the approved questions and EHR retrieval tasks to generate a complete reasoning response to each question including all EHR variables (Fig. 8C).

For refinement of the ICU-REACT train seed set, clinician comments were first classified by the refinement agent into three feedback types: variable selection feedback, reasoning improvement feedback, and context-question improvement feedback, with remaining comments tagged as 'unclassified' (Fig. 8C). Variable selection feedback captured issues related to missing, irrelevant, or incorrectly mapped EHR variables; reasoning improvement feedback identified gaps in clinical logic, completeness, or interpretability; and context-question improvement feedback described concerns about the clarity or alignment of the patient context and decision-making question.

We focused on feedback related to variable selection and reasoning improvement, as these directly addressed the model outputs used for clinical reasoning supervision. Each refinement sample was constructed from the original context-question pair and the initial reasoning generated by the GPT-4.1-based Reasoning Agent described in Section 4.1.1. This information, together with the clinician feedback, was provided to the refinement agent, which was prompted to produce two outputs: an explanation of how the initial reasoning should be improved and a corresponding improved reasoning response, both aligned with the clinician feedback (Fig. 8C).

The train seed was then consolidated as samples containing the patient context, decision-making question, initial reasoning, explanation of the needed reasoning improvements, and improved reasoning (Fig. 8C). This structure preserved the original model rationale while adding clinician-guided supervision that explicitly teaches how clinical reasoning should be revised, providing the curated foundation for subsequent data augmentation.

#### 4.1.2 Dataset augmentation

Following the construction of the ICU-REACT train seed dataset, we implemented an automated augmentation pipeline to expand the dataset's size to allow for model training (Fig. 9).

The objective of the augmentation process was to increase the diversity of clinical reasoning examples while maintaining the structure, terminology, and expert-aligned reasoning schema established in the original seed dataset. To achieve this, a GPT-5.2 augmentation agent was used to generate the new context-question pairs through carefully controlled prompting. Specifically, a clinician-guided ICU topic list was used to iteratively prompt the augmentation agent to produce diverse patient contexts and

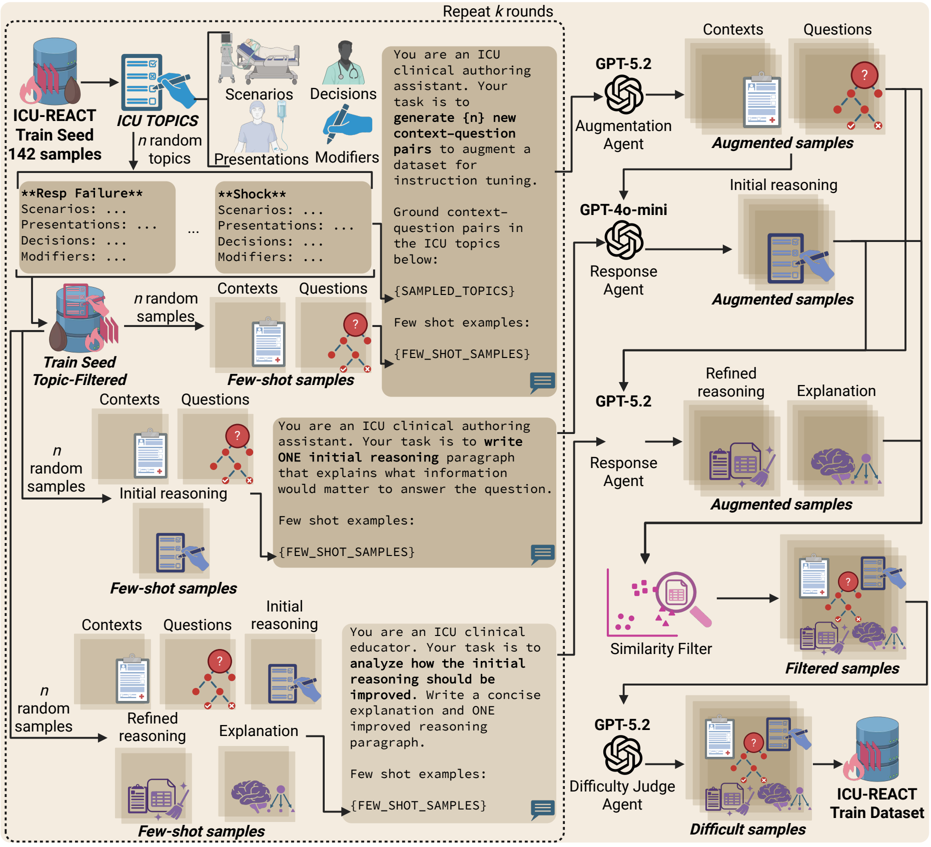

**Fig. 9** Dataset augmentation overview. Beginning with the clinician-validated ICU-REACT-Train seed set, the pipeline iteratively sampled ICU topics together with topic-specific scenarios, patient presentations, clinical modifiers, and decision types to guide generation of diverse context–question pairs. At each generation stage, few-shot examples were randomly sampled from the train seed dataset and incorporated into the corresponding prompts to preserve the expert-aligned structure, terminology, and reasoning schema of the seed examples. A GPT-5.2 augmentation agent generated new patient contexts and ICU decision-making questions, after which a GPT-4o-mini agent produced initial reasoning responses. A GPT-5.2 refinement agent then generated a refinement explanation and an improved reasoning response from the initial reasoning, using additional seed-derived few-shot examples to maintain consistency with clinician-validated reasoning patterns. To reduce redundancy, augmented samples were vectorized, grouped through nearest-neighbor search, and compared within groups using ROUGE-L; samples with ROUGE-L scores of at least 0.7 were removed. Finally, a GPT-5.2 difficulty-judging agent categorized retained samples as easy, medium, or hard, and samples were filtered according to difficulty level to construct training sets tailored to each model architecture and scale, producing the final ICU-REACT-Train datasets.

decision-making questions, by sampling $n$ topics at each augmentation iteration. To further improve diversity of the dataset, we used ChatGPT to create lists of patient scenarios, presentations, modifiers and decisions within each ICU topic, sampling $n$ items of each category at each augmentation iteration. The full list of topics with their corresponding scenarios, presentations, modifiers and decisions can be found on

Supplemental Section S12. Following this, a GPT-4o-mini agent was used to generate initial reasonings for each augmented pair, and subsequently, a GPT-5.2 agent was used to generate refined reasonings along with explanations for the refinement based on the initial reasonings, mirroring the schema and structure of the clinician-validated train seed dataset. At each generation stage, we randomly sampled few-shot examples from the ICU-REACT train seed dataset and incorporated them into the corresponding prompts to preserve the expert-aligned structure, terminology, and reasoning schema.

To reduce redundancy among augmented samples, we applied a similarity filter by vectorizing samples and grouping them using nearest-neighbor search. Samples within each group were then compared pairwise using ROUGE-L, and samples with a ROUGE-L score of 0.7 or higher were removed. Finally, we further filtered samples by difficulty level. Specifically, a GPT-5.2 difficulty judge agent was used to classify samples in one of three difficulty categories: easy, medium, and hard. Subsequently, samples were filtered according to difficulty level to construct training sets tailored to each model architecture and scale, producing the final ICU-REACT Train Datasets.

### 4.2 Model training

We developed Clin-REACT, a set of fine-tuned LLMs for decision-focused clinical reasoning and information retrieval in the ICU. Clin-REACT models were initialized from Llama, Gemma, and Baichuan base models and trained on the ICU-REACT Train Datasets using supervised fine-tuning (SFT) (Fig. 10A).

#### 4.2.1 Base models

We trained Clin-REACT variants from four base models: Llama 3.1 8B, Llama 3.3 70B, Gemma 4 31B, and Baichuan M1 14B. These models span different architectures and parameter counts, allowing to assess the robustness of ICU-REACT as a training dataset for improving decision-focused clinical reasoning beyond a single model family and size. Training multiple Clin-REACT variants also provided a set of models with different size-performance tradeoffs, supporting evaluation across both more efficient and higher-capacity deployment settings.

#### 4.2.2 Training strategy

The models were trained with an assistant-only causal language modeling objective through parameter-efficient fine-tuning (PEFT). We used low-rank adaptation (LoRA) to fine-tune each base model by freezing the original model weights and learning small trainable low-rank update matrices for selected linear layers [37]. For a pretrained weight matrix $W_0 \in \mathbb{R}^{d\times k}$, LoRA parameterized the adapted weight as:

$$W = W_0 + \Delta W = W_0 + \frac{\alpha}{r} BA,$$

where $A \in \mathbb{R}^{r\times k}$ and $B \in \mathbb{R}^{d\times r}$ are trainable low-rank matrices, $r \ll \min(d, k)$ is the LoRA rank, and $\alpha$ is a scaling factor that controlled the magnitude of the learned update. During training, the pretrained weights $W_0$ remained fixed, and only

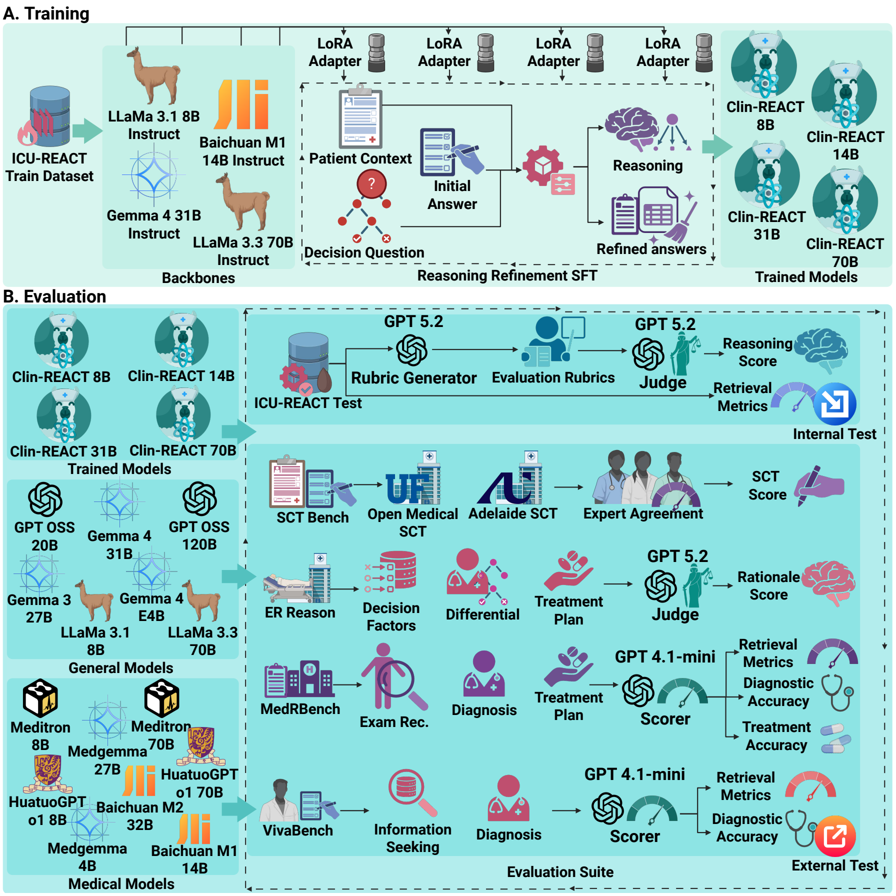

**Fig. 10** Model Training and Evaluation. (A) Supervised fine-tuning of four open-source backbone models using the ICU-REACT-Train datasets. Low-rank adaptation (LoRA) adapters were trained through reasoning-refinement supervision, with the patient context, decision-making question, and initial response provided as model inputs, and the refinement explanation and improved reasoning response used as target outputs. This process yielded Clin-REACT 8B, 14B, 31B, and 70B models. (B) Evaluation of Clin-REACT models alongside open-source general-purpose and medical LLM baselines. In-domain performance was assessed on the held-out ICU-REACT-Test set using automated variable-retrieval precision, recall, and F1 metrics, together with GPT-5.2 rubric-based reasoning scores. External evaluation included SCT-Bench, which measured agreement with expert response distributions under clinical uncertainty; ER-Reason, which evaluated precision and recall of clinically relevant concepts in acute-care rationales; MedRBench, which assessed examination recommendation, diagnostic, and treatment performance; and VivaBench, which evaluated information-seeking and final diagnostic accuracy in multi-turn clinical cases.

the LoRA parameters $A$ and $B$ were optimized, substantially reducing the number of trainable parameters while adapting the model to ICU-REACT reasoning refinement.

Each training example consisted of a patient context $c$, a decision-making question $q$, an initial reasoning response $r^{(0)}$, and a target assistant response $a$. The target response $a$ corresponded to the reasoning refinement and contained two components: an explanation of how the initial reasoning should be improved and the final improved reasoning. The models were optimized only on the assistant response tokens, while the input tokens from the context, question, and initial reasoning were used as conditioning information:

$$\mathcal{L}_{\text{refine}}(\theta) = -\sum_{i=1}^{N}\sum_{t=1}^{|a_i|}\log P_\theta\Big(a_{i,t} \mid a_{i,<t}, c_i, q_i, r_i^{(0)}\Big)$$

where $N$ is the number of training examples, $c_i$ denotes the patient context for example $i$, $q_i$ denotes the corresponding decision-making question, and $r_i^{(0)}$ denotes the initial reasoning generated before refinement. The target assistant response $a_i = (a_{i,1}, \dots, a_{i,|a_i|})$ is the reasoning refinement sequence, consisting of both the explanation of the needed refinement and the improved reasoning. The term $a_{i,t}$ denotes the $t$-th target token, $a_{i,<t}$ denotes all preceding assistant-response tokens, and $P_\theta$ is the conditional token probability assigned by the model with parameters $\theta$. Because the loss was computed only over the assistant response tokens $a_i$, the model learned to use the patient context, decision-making question, and initial reasoning as input while being supervised to generate the refined reasoning output.

### 4.3 Model evaluation

We evaluated Clin-REACT models and compared their performance against open-source general-purpose and medical LLMs on internal and external benchmarks to measure their performance on clinical reasoning (Fig. 10B).

#### 4.3.1 Benchmarks

We evaluated Clin-REACT on five clinical reasoning benchmarks that probe ICU-specific reasoning, general clinical reasoning, and acute care decision-making:

- **ICU-REACT Test (in-domain) (n = 71).** A held-out test set drawn from the ICU-REACT distribution, used to measure in-domain generalization for both reasoning generation and structured variable retrieval. This benchmark directly reflects the target use case of ICU decision support. For evaluation, we measured automated retrieval metrics (i.e., precision, recall and F1 scores) for variable domains/categories and individual variables, as well as LLM-judge-based reasoning score using LLM-generated evaluation rubrics. Specifically, we used GPT-5.2 to first generate evaluation rubrics for each sample on the test dataset following similar methodologies from previous literature [38]. We then used a GPT-5.2-based judge which scored model responses strictly following the evaluation rubrics. The final reasoning score was calculated from adding all points given on each rubric criteria and normalizing the score based on the maximum possible number of criteria points.
- **SCT-Bench (n = 174).** [20] A script concordance test (SCT)-style benchmark assessing clinical reasoning under uncertainty. Specifically, we used the public set of this benchmark which contains the Open Medical SCT from the University of Florida and the Adelaide SCT from Adelaide University. Performance was computed using the SCT scoring procedure that rewards alignment with expert response distributions, emphasizing calibration rather than single-label correctness.

- **ER-Reason (n = 72).** [23] An emergency medicine reasoning benchmark evaluating acute care decision-making and prioritization in time-sensitive settings, providing an out-of-domain but clinically adjacent stress test. We focused on the rationale module of this dataset, which contains physician-written explanations for decision factors, differential diagnosis, and treatment planning across 72 emergency room cases. To evaluate model outputs, we used a GPT-5.2-based LLM-judge approach to assess the quality of each generated rationale by quantifying recall and precision of relevant medical concepts present in the original physician-written rationales for each category.
- **MedRBench (n = 1,338).** [21] A reasoning-focused benchmark built from publicly-available structured real-world clinical case reports, evaluating models across examination recommendation, diagnostic decision-making, and treatment planning, with explicit assessment of final clinical outputs. Prior to evaluation, we excluded samples that triggered Azure content-policy filters during processing with Azure-hosted GPT helper or judge models, and used the resulting filtered set consistently across all evaluated models to ensure an identical evaluation cohort. Performance of all models was measured following the same methodology as in the original paper [21], using GPT-4.1-mini instead of GPT-4o as the helper and judge model due to budget constraints. We focused on examination recommendation recall and precision, diagnosis accuracy and treatment accuracy.
- **VivaBench (n = 934).** [22] A multi-turn benchmark that simulates viva voce-style clinical examinations built using clinical vignettes from publicly available repositories, requiring models to iteratively gather relevant history, physical examination findings, and diagnostic investigations before synthesizing a final diagnosis. Prior to evaluation, we excluded samples that triggered Azure content-policy filters during processing with Azure-hosted GPT helper or judge models, and used the resulting filtered set consistently across all evaluated models to ensure an identical evaluation cohort. Performance of all models was measured following the same methodology as in the original paper [22], using GPT-4.1-mini instead of GPT-4.1 as the mapper and judge model due to budget constraints. We focused on information seeking recall and precision, and diagnosis accuracy.

Together, these benchmarks quantified (i) in-domain performance on ICU decision-making and variable retrieval (ICU-REACT Test), (ii) robustness to uncertainty and partial information (SCT-Bench and VivaBench), and (iii) generalization to broader clinical reasoning and acute-care workflows (ER-Reason and MedRBench).

#### 4.3.2 Baselines

We compared Clin-REACT model variants against both open-source general-purpose LLMs and medical-domain LLMs. The general-purpose comparison models included GPT-OSS models (GPT-OSS 20B and GPT-OSS 120B) [39], Gemma models (Gemma 3 27B and Gemma 4 31B) [29], and Llama models (Llama 3.1 8B Instruct and Llama 3.3 70B Instruct) [28]. The medical-domain comparison models included

Meditron models (Meditron 3 8B and Meditron 3 70B) [40], MedGemma models (MedGemma 4B and MedGemma 27B) [16], HuatuoGPT-o1 models (HuatuoGPT-o1 8B and HuatuoGPT-o1 70B) [17], and Baichuan models (Baichuan M1 14B Instruct and Baichuan M2 32B) [18, 19]. This comparison set was designed to contextualize Clin-REACT performance against models spanning different parameter scales, model families, and degrees of medical specialization.

#### 4.3.3 Statistical Analyses

We performed statistical analyses to compare Clin-REACT model variants against zero-shot baseline models across the five clinical reasoning benchmarks. Because all models were evaluated on the same benchmark instances within each dataset, model comparisons were treated as paired analyses. All benchmark metrics operated on the $[0, 100]$ range, with higher scores indicating better performance.

A non-parametric bootstrap procedure with 1000 iterations was employed to estimate the mean and 95% confidence intervals of the evaluation metrics across each benchmark. In each iteration, a resampled dataset equal in size to the test set of the benchmark being evaluated was generated via random sampling with replacement.

For pairwise model comparisons, statistical significance was assessed using the two-sided Wilcoxon signed-rank test. For a given comparison, we calculated the paired difference in score between the two models across benchmark instances and tested whether the median paired difference differed from zero.

## 5 Data availability

The ICU-REACT dataset, including the seed training set, the ICU-REACT-Train-Small, Medium, and Large augmented training sets, and the held-out ICU-REACT-Test benchmark with its sample-specific evaluation rubrics, is publicly available through Hugging Face at https://huggingface.co/datasets/macontreras98/ICU-REACT. The external clinical-reasoning benchmarks evaluated in this study are available from their respective original sources. SCT-Bench is publicly available at https://github.com/SCT-Bench/sctpublic; MedRBench is publicly available at https://github.com/MAGIC-AI4Med/MedRBench; and VivaBench is publicly available through Hugging Face at https://huggingface.co/datasets/chychiu/VivaBench. ER-Reason is available through PhysioNet at https://physionet.org/content/er-reason/1.0.0/. Access to ER-Reason requires PhysioNet credentialing, completion of the Collaborative Institutional Training Initiative (CITI Program) "Data or Specimens Only Research" training module, agreement to the applicable data use agreement, and approval of a project-specific access request by the data contributors.

## 6 Code availability

The code for dataset processing and augmentation, model training and inference, benchmark evaluation, and all analyses reported in this study is freely available at https://github.com/iheallab/icureact. The trained Clin-REACT model checkpoints are publicly available through Hugging Face: Clin-REACT 8B at https://

huggingface.co/macontreras98/Llama-Clin-REACT-8B, Clin-REACT 14B at https://huggingface.co/macontreras98/Clin-REACT-14B, Clin-REACT 31B at https://huggingface.co/macontreras98/Clin-REACT-31B, and Clin-REACT 70B at https://huggingface.co/macontreras98/Llama-Clin-REACT-70B. The model checkpoints are distributed subject to the respective licenses and terms of their underlying base models.

## 7 Acknowledgments

A.B and P.R. were supported by NIH/NINDS R01 NS120924 and NIH/NIBIB R01 EB029699.

## Supplementary Files

This is a list of supplementary files associated with this preprint. Click to download.

- supplement.pdf
